# Clinical, Imaging and Histopathology of Angioleiomyoma of the Buccal Cheek

**DOI:** 10.1155/2021/9940304

**Published:** 2021-04-30

**Authors:** Mohammad Jafarian, Fatemeh Mashhadi Abbas, Mitra Ghazizadeh Ahsaie, Homeira Saebnoori

**Affiliations:** ^1^Department of Oral and Maxillofacial Surgery, School of Dentistry, Shahid Beheshti University of Medical Sciences, Tehran, Iran; ^2^Department of Oral and Maxillofacial Pathology, School of Dentistry, Shahid Beheshti University of Medical Sciences, Tehran, Iran; ^3^Department of Oral and Maxillofacial Radiology, School of Dentistry, Shahid Beheshti University of Medical Sciences, Tehran, Iran

## Abstract

Angioleiomyoma is a benign neoplasia originating from vascular smooth muscle and very uncommon in the oral cavity. In this report, we present a rare case of angioleiomyoma in oral cavity in a 46-year-old female buccal cheek and discuss the clinical, histological, and immunohistochemical characteristics. As the treatment of choice is the complete excision, the lesion was excised under local anesthesia with no further complications. In addition, a brief update on other reported cases of angiomyoma in the oral cavity is further discussed.

## 1. Introduction

Angiomyoma or angioleiomyoma (also known as vascular leiomyoma) are benign neoplasms originating from vascular smooth muscle [1, 2]. Histologically, there are subgroups in three categories: solid, venous, and cavernous [3]. They commonly occur in the extremities and female genital tract and rarely in the oral cavity [4, 5]. The most common site in the oral cavity is in the lips [1]. Other reported sites were the palate, tongue, cheek, gingiva, retromolar area, and submandibular triangle [2, 6–10]. Although the etiology is still unclear, previous studies report trauma or spontaneous development as the causes of origin in oral cavity [10, 11]. The lesion is most often detected in adults of 30 to 50 years old [3]. Clinically, it usually presents as a slow growing, painless nodule or mass of variable size; however, pain has also been reported [12]. The final treatment and diagnosis is based on surgical excision and hematoxylin and eosin (H&E) staining and immune-histochemical (IHC) assessments [1]. In this report, a rare case of angioleiomyoma in the cheek along with a complete imaging assessment including color Doppler ultrasonography, computed tomography (CT), and magnetic resonance imaging (MRI) with an update on the literature is presented.

## 2. Case Report

**Figure 1:**
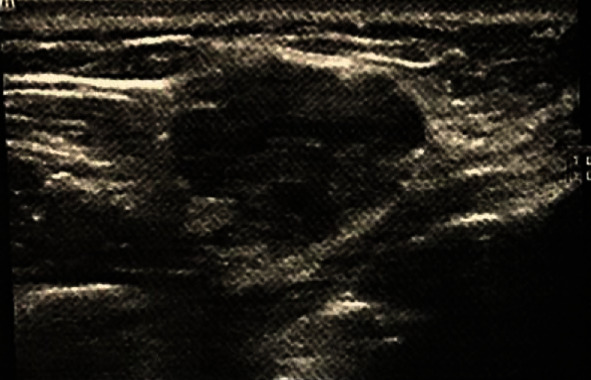
Ultrasonography shows a well-defined hypoechoic heterogenous mass lying between the skin and buccinator muscle.

**Figure 2:**
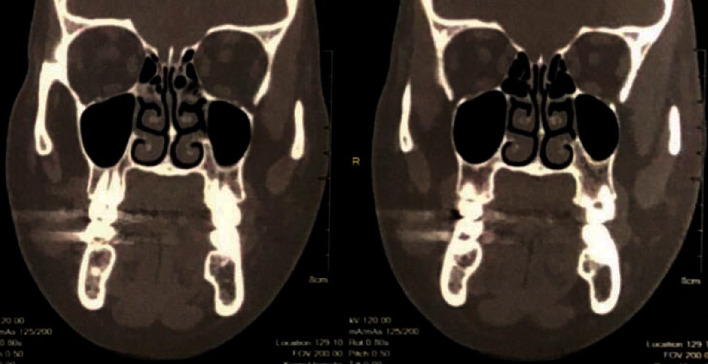
Coronal MDCT image shows a circular well-defined homogenous mass in the right buccal area.

**Figure 3:**
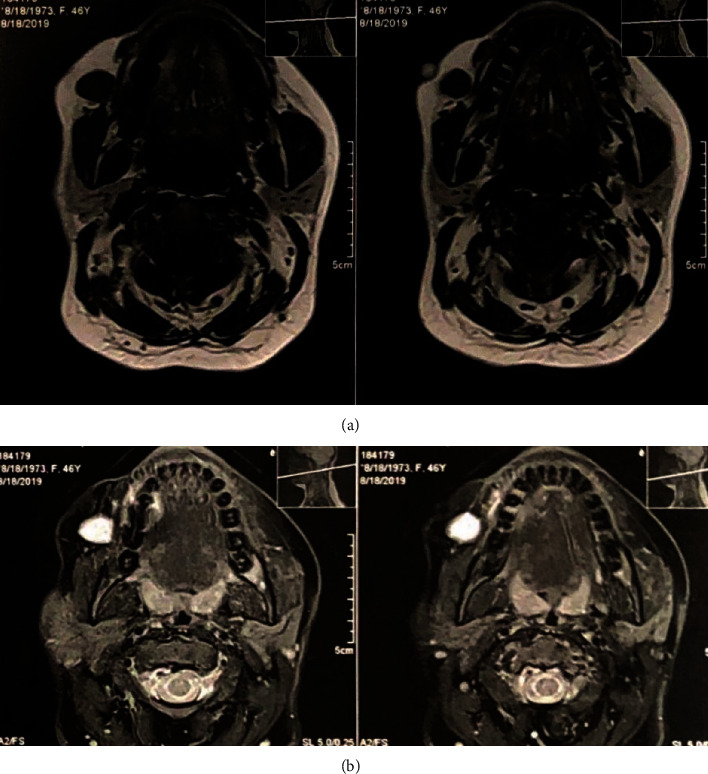
(a) Axial T1 MRI shows a low signal homogenous mass in the right lying over the buccinator muscle and under the skin. (b) Axial T2 MRI shows the lesion as high signal and homogenous.

**Figure 4:**
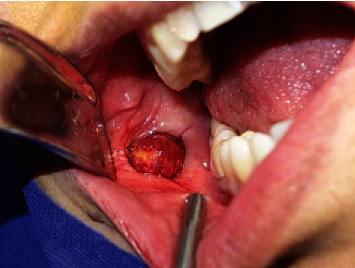
Intraoral incision on the buccal mucosa revealed a spherical lesion just underneath the buccinator muscle.

**Figure 5:**
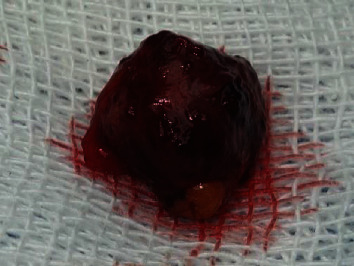
Image shows gross specimen, a spherical brown-gray firm tissue, measuring 1 cm in diameter.

**Figure 6:**
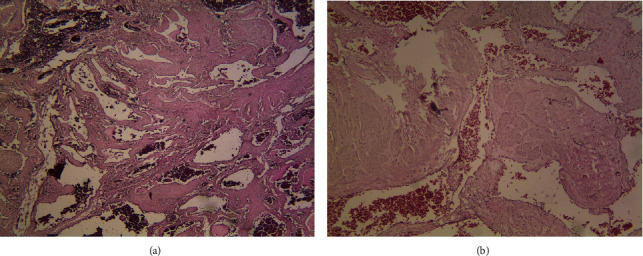
(a) Thin blood vessels with papillary projections in the fibrous stroma (×40). (b) Paralleling pattern of fascicle and glomus cells around the blood vessels (×100).

**Figure 7:**
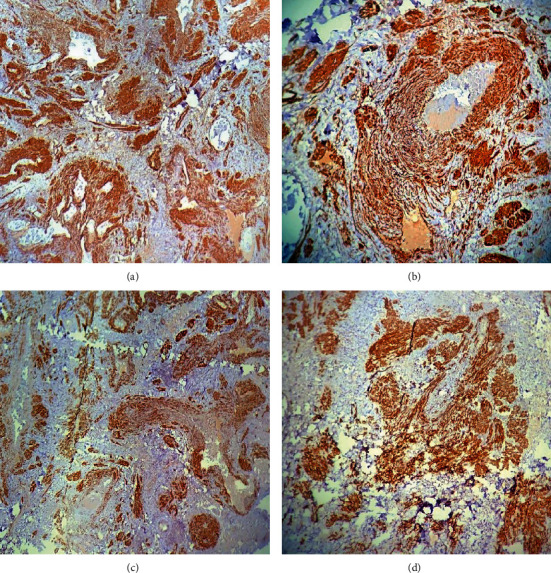
(a) ×40 and (b) ×100 positive IHC staining for SMA. (c) ×40 and (d) ×100 positive for desmin.

A 46-year-old female was referred to a private clinic of an Oral and Maxillofacial Surgeon with complaint of a painless swelling in the right side of her cheek since 6 years ago. The swelling was initially small in size and slowly enlarged, although the patients faced no tenderness in the area. The patient had diabetes mellitus and anemia. In addition, no history of previous trauma to the area was present. Extraoral examination showed no asymmetry or marked expansion on the right cheek. On intraoral examination, no clinically visible lesion was detected. The mucosa was intact with no color change. When the area was palpated, a firm, compressible solid mass was detected with moderate attachment to the adjacent tissues. The estimated size of the lesion was 1.5∗1 cm, and the approximate diameter was 1.5 cm. No pulsation or bruit was present. The patient had previously visited and had prior imaging including Doppler ultrasonography, multidetector computed tomography (MDCT), and MRI. The results of ultrasonography indicated a well-defined hypoechoic heterogeneous mass lying beneath the skin measuring 13∗18 mm (Figure 1). The lesion had moderate vascular flow. MDCT showed a circular well-defined homogenous mass over the buccinator muscle (Figure 2). The MRI T1 sequence showed a well-defined low signal mass between the buccinator muscle and buccal fat (Figure 3(a)). The MRI T2 sequence showed that the mass is homogenous and high signal (Figure 3(b)). A soft tissue mass was suspected, and an intraoral surgical excisional biopsy was planned. Upon obtaining consent, an excisional biopsy was performed under local anesthesia and incision was made on the buccal mucosa just over the palpated mass (Figure 4). The lesion was completely excised (Figure 5). H&E staining histopathological examination demonstrated an encapsulated mass composed of fully developed cavernous angioma in a fibrous stroma and occasionally myxomatous change. The thin-wall blood vessels showed papillary projections to the lumen which some had become irregular, large in size, and even sinusoid in some areas (Figure 6(a)). The stroma contains immature spindle-shaped fibroblasts with a paralleling fascicle pattern next to the blood vessels (Figure 6(b)). The mixed inflammatory infiltration and hyalinized area intermixed with adipose tissue were also found. In the IHC staining analysis, the specimen was positive for both SMA (smooth muscle actin) (Figures 7(a) and 7(b)) and desmin (Figures 7(c) and 7(d)); however, the microscopic features in combination with IHC findings were consistent with angioleiomyoma diagnosis. A postoperative follow-up of the patient was uneventful. The area had healed with no complication, and no signs of recurrence were detected.

## 3. Discussion

Angioleiomyoma is a rare benign soft tissue neoplasm of oral cavity [7]. Although hormonal changes, trauma, and venous stasis are suggested as possible causes, the etiology is still unknown [5]. On our latest review of literature from year 2000 to 2020 using the PubMed Central search engine, 25 articles (32 cases) were retrieved (Table 1). The most common sites of oral angiomyomas were the lip [2, 4, 12–15], cheek or buccal mucosa [5, 8, 14, 16–18], palate [7, 19–21], tongue [6, 22, 23], and submandibular area [9, 11, 24]. The gingiva [10], mandible [2], retromolar area [2], and anterior maxillary labial fold [17] were each reported once among the studies. 73% of patients were male, and 27% were females, which is consistent with previous studies [25, 26]. The mean age was 43 ± 16.57 years old (range 2 to 79). Only one study reported a congenital angiomyoma in the tongue [3]. In all reviewed studies, the patient faced a painless swelling. Only three cases reported pain associated with the lesion [7, 21, 24].

Preoperative radiologic assessments varied among the studies, from no radiographic assessments and plain radiography to ultrasound, MRI, and CT evaluations. To our knowledge, this is the first case report of angioleiomyoma with a thorough imaging assessment including color Doppler sonography, MDCT, and MRI. The lesion was slightly heterogeneous hypoechoic mass with slight vascular flow in the color Doppler sonography; in addition, the T1-weighted and T2-weighted sequences were low and high, respectively. The high T2-weighted sequence may be seen in cysts, benign or low-grade minor salivary gland tumors, and rare hemangiomatous lesions [27]. In this case, differential diagnosis such as benign lesions of salivary gland origin was suggested in lower possibility because the lesion was located between the buccinator muscle and skin. Cystic lesions were also excluded as the sonography of the cyst is homogenous and unechoic [28]. Differential diagnosis may suggest other benign mesenchymal tumors such as fibroma, lipoma, and neurofibroma and vascular lesions including arteriovenous malformation, lymphangioma, and hemangioma. However, these lesions may have different radiographic and imaging characteristics. According to previous studies, the intraosseous angioleiomyomas are radiographically unilocular or multilocular radiolucent lesions. They can have either an ill-defined or a well-defined sclerotic border [2, 11].

In this case, H&E histologic examination showed a tumor consisting of thin- and thick-walled blood vessels in a background of smooth muscle proliferation. Having numerous blood vessels may pretend other benign vascular tumors such as hemangioma, hemangiopericytoma, hemangioendothelioma, vascular malformation, and other neurovascular hamartomas [29], but a definite examination could rule out these lesions because of the smooth muscle background. Immunohistochemically markers like SMA and MSA (muscle-specific actin) can be useful in identification of smooth muscle nature of the cellular stroma [15, 30, 31]. In the present study, IHC staining was positive for both desmin and SMA. Various IHC stainings were carried out among studies (Table 1) although SMA and desmin were the most frequent. Depending on the apparent features of the blood vessels, IHC staining of endothelial cell markers such as CD34 and CD31 was not recommended. Other differential diagnosis histopathologically is leiomyosarcoma if there were more cellular pleomorphism and mitosis figures. In addition to well circumscribing of this tumor, lacking of anaplasia and bizarre cells, fewer than 5 mitoses per 20 high-power fields could rule out sarcoma.

In all evaluated cases, the lesion was resected with an excisional biopsy under local or general anesthesia. There are no recurrences after resection.

## 4. Conclusion

In conclusion, we have reported a case of angioleiomyoma of the buccal cheek that resulted in a good outcome. Among various studies, the most common sites of oral angiomyomas were the lip [2, 4, 12–15, 32], cheek or buccal mucosa [5, 8, 14, 16–18], palate [7, 19–21], tongue [6, 22, 23], and submandibular area [9, 11, 24]. Other areas such as the gingiva, mandible, retromolar area, and anterior maxillary labial fold were only reported once among studies [2, 10, 17]. Due to the benign nature of this lesion, the treatment is excisional biopsy and there have been no recurrences or complications reported so far [32].

## Figures and Tables

**Table 1:** Review of various studies on angioleiomyoma of head and neck. cm: centimeter; CT: computed tomography; D: day; F: female; H&E: hematoxylin and eosin; IHC: immunohistochemistry; M: male; Mo: month; mm: millimeter; MRI: magnetic resonance imaging; SMA: smooth muscle actin; Y: year; YO: years old.

No	Author & year	Age & sex	Location	Size	Sign & symptoms	Radiologic assessments	Treatment plan	Anesthesia	Histologic assessments	Follow-up
1	Mehta et al., 2020 [15]	57 YO, M	Lower lip	1 × 1 cm	Exophytic nodular swelling	—	Surgical excision	Local	H&E: Large vascular channels of varying caliber, surrounded by thick walls of irregularly arranged spindle-shaped cells. Whorled bundles of muscle fibers were seen fused with the vessel walls IHC: SMA+	3 Mo, no recurrence
2	Giudice et al., 2019 [32]	17 YO, F	Cheek	1.5 × 1.0 cm	Swelling	—	Surgical excision	Local	H&E: Uniform spindle smooth muscle cells with pale eosinophilic cytoplasm, low amount of fibrous connective tissue, and distributed around numerous small tortuous “slit-like” vessels IHC: SMA+ CD34+ Desmin+ Vimentin+	1 week, 4 weeks, 6 months No recurrence
3	Pandya et al., 2019 [23]	46 YO, F	Palate	1.5 × 1.5 × 0.5 cm	Exophytic growth, pain	Occlusal plain film	Surgical excision	Local	H&E: Numerous thick-walled blood vessels in the connective tissue formed of hyperplastic smooth muscle fibers IHC: SMA+	6 Mo, no recurrence
4	Perić et al., 2019 [4]	36 YO, M	Lip	5 × 3 mm	Painless swelling	—	Surgical excision	Local	H&E: Numerous thick-walled vessel IHC: CD34+ SMA+	7 D, normal healing
5	Ribeiro et al., 2019 [18]	60 YO, M	Anterior maxillary labial fold	—	Swelling	—	Surgical excision	—	H&E: Multiple vessels, thickened muscular layer IHC: CD34+ SMA+	2 Y, no recurrence
6	Ribeiro et al., 2019 [18]	33 YO, M	Buccal mucosa		Submucosal nodule	Color Doppler ultrasonography: well-limited hyperechoic area between cutaneous and muscular layers, no change in blood flow	Surgical excision		H&E: Multiple vascular spaces of various sizes and calibers, sometimes congested and interconnected IHC: CD34- SMA+	
7	S.Y. Rawal and Y.B. Rawal, 2018 [22]	70 YO, M	Hard palate	2.0 × 1.5 cm	Painless mass	—	Surgical excision	—	H&E: Brightly eosinophilic, spindle-shaped cells IHC: SMA+	—
8	Hassona et al., 2017 [14]	52 YO, F	Upper lip	—	Painless mass	—	Surgical excision	Local	H&E: Vascular channels surrounded by fascicles of concentrically arranged spindle cells with eosinophilic cytoplasm IHC: SMA+	12 Mo, no recurrence
9	Osano et al., 2015 [5]	45 YO, M	Cheek	22 × 15 × 11 mm	Painless mass	MRI: T1+contrast: homogeneously enhanced mass and a vessel leading to the tumor T2: Uniform signal and hyperintense signal	Surgical excision	General	H&E: Proliferation of vessels with a smooth muscle wall and vessels surrounded by smooth muscle cells IHC: SMA+ Desmin+ Vimentin+	18 Mo, no recurrence
10	Ishikawa et al., 2014 [6]	51 YO, M	Tongue	11 mm	Nodular mass	—	Surgical excision	—	H&E: Brightly eosinophilic, spindle-shaped cells IHC: SMA+ HHF-35 actin+ Vimentin+ Desmin+ S100- CD34-	8 Mo, no recurrence
11	Tsuji et al., 2014 [7]	79 YO, M	Hard palate	1.5 × 1.5 cm	Painless swelling	Panoramic: No source of odontogenic infection CT: Well-circumscribed mass, no evidence of surrounding bone resorption	Surgical removal and curettage	Local	H&E: Vascular spaces and redundant smooth muscle IHC: Desmin+ SMA+	6 Mo, no recurrence
12	Eley et al., 2012 [21]	39 YO, M	Hard palate	2 cm	Painless swelling	MRI: T1: Slightly higher than the surrounding soft tissue T2: Marked hyper intensity	Surgical excision	General	H&E: Vascular channels surrounded by fascicles of spindle cells IHC: Desmin+ SMA+	3 Mo, no recurrence
13	Minni et al., 2012 [11]	54 YO, F	Submandibular space	3 × 3 cm	Painless swelling	MRI: Displacement of submandibular gland, tongue, and oropharynx airway Compressing the right tonsil Ultrasound-guided FNA: Blood-stained aspirate	Complete surgical removal	General	H&E: Smooth muscle tissue punctuated with thick-walled vessels, capillary, and venous-type vessels IHC: SMA+	6 Mo, no recurrence
14	Menditti et al., 2012 [10]	14 YO, M	Gingiva	1-2 cm	Painless swelling	—	Radical excision with 2 mm free margins	Local	H&E: Vascular spaces and redundant smooth muscle IHC: SMA+	1 Y, no recurrence
15	Gueiros et al., 2011 [16]	-54 YO, M -66 YO, M -53 YO, M	Lower lip Upper lip Upper lip	−1 × 1 cm −0.8 × 0.5 × 0.5 cm	Painless nodule	—	Surgical excision	Local	H&E: Spindle-shaped cells and blood vessels IHC: MSA+	-2 Y -1 Y -6 Mo, no recurrence
16	Vidaković et al., 2011 [8]	58 YO, M	Cheek	2 × 1.5 cm	Painless swelling	—	Surgical excision	Local	H&E: Thick-walled vessels with partially patent and smooth muscle fibres	7 D, normal healing of wound
17	Kim et al., 2010 [3]	2 YO, M	Tongue	2.5 × 2 cm	Soft tissue mass	MRI: Elevated mass on the base of the tongue T1: Isointense signal to muscle T2: Slightly hyperintense signal with hyperintense rim	Surgical removal	General	H&E: Prominent and thickened vessel walls consisting of benign and mature smooth muscle cells IHC: Desmin+ SMA+	26 Mo, no recurrence
18	Nonaka et al., 2010 [25]	38 YO, M	Tongue	2 cm	Painless exophytic mass	—	Surgical excision	—	H&E: Proliferation of spindle-shaped nucleus cells, numerous blood vessels IHC: SMA+	1 Y, no recurrence
19	Keerthi et al., 2009 [17]	32 YO, M	Cheek	4.5 × 4 cm	Painless swelling	CT: Large nonhomogenously enhancing mixed density lesion in the infratemporal fossa and the buccal space, moderately enhancing soft tissue component superolaterally, with no calcification or necrosis	Surgical removal	General	H&E: Multiple blood vessels with proliferation of smooth muscle	6 Y, no recurrence
20	Keerthi et al., 2009 [17]	32 YO, M	Cheek	3 × 3 cm	Painless swelling	Plain film radiograph: No bony changes Ultrasound: Hypoechoic lesion more in favor of soft tissue swelling	Surgical removal	General	H&E: Spindle-shaped fascicles and many blood vessels IHC: Masson trichrome stain (+)	1 Y, no occurrence
21	McParland et al., 2009 [19]	42 YO, M	Buccal mucosal	3 × 3 cm	Painless swelling	—	Surgical excision	Local	H&E: Smooth muscle bands are surrounded by slit-like vascular spaces	6 Mo, no recurrence
22	Cepeda et al., 2008 [2]	39 YO, F 27 YO, F 43 YO, F 36 YO, M 48 YO, M	Retromolar area Mandibular region Lower lip Upper lip Upper lip	0.9 × 0.6 × 1 cm 1.4 × 1.3 × 1 cm 0.7 × 0.5 × 0.4 cm 1.5 × 1.0 × 1.0 cm 1.7 × 1.0 × 1.0 cm	Painless mass	Routine radiographic inspection: A unilocular radiolucency located in a mandibular region (in case 2)	Surgical excision	—	H&E: Several blood vessels lined by a thin layer of endothelial cells were observed intercalated in the fascicules IHC: SMA+ Vimentin+ Desmin+ CD34-	
23	Manor et al., 2007 [20]	39 YO, M	Buccal vestibule	8	Asymptomatic mass	CT: Hypervascular soft tissue mass, with no invasion of the periosteum or bone	Surgical excision	—	H&E: Bands of smooth muscle cells surrounding multiple vascular spaces of varying size IHC: SMA+	12 Mo, no recurrence
24	Ide et al., 2003 [26]	40 YO, F	Submandibular gland	2.0 × 1.8 × 1.5 cm	Painful swelling	—	Surgical excision		H&E: Tortuous thick-walled vessels in varying sizes, proliferation of smooth muscle IHC: SMA+ CD31– CD34– S-100-	
25	Toida et al., 2000 [12]	10 YO, M	Lower lip	2 × 1.5 cm	Painful mass	—	Surgical excision	Local	H&E: Spindle cells surrounded by numerous slit-like vessels IHC: SMA+ S-100-	3 Y, no recurrence
26	Simon et al., 2000 [9]	59 YO, F	Submandibular triangle	15 × 13 mm	Nontender mass	Color Doppler ultrasound: Well-circumscribed, homogeneous solid mass attached to the posterior surface of the submandibular gland, vascularity within the mass	Surgical excision	General	H&E: Smooth muscle punctuated with thick-walled venous vascular channels	—

## Data Availability

The data used to support the findings of this study are available from the corresponding author upon request.
